# Reviewing the Use of a Fibroscan® Machine in Belfast Trust Addictions Service

**DOI:** 10.1192/bjo.2024.482

**Published:** 2024-08-01

**Authors:** Benjamin Johnston, Helen Toal, Joy Watson

**Affiliations:** Belfast H&SC Trust, Belfast, United Kingdom

## Abstract

**Aims:**

Belfast Trust Addictions Service was among the first addictions teams in the UK to get their own Fibroscan® machine, in March 2021. In the two preceding years (2019–2020), only 32% of patients referred by addictions to hepatology for hepatitis C virus (HCV) attended their appointments.

Patients under the addictions service are known to access healthcare services poorly while being at increased risk, with a clear need to improve their access to appropriate care.

We aimed to review how the Fibroscan® machine has been used in the addictions service, and if there has been an impact on how the patient cohort access healthcare.

**Methods:**

We reviewed our case records of all patients offered a Fibroscan®, and whether they attended the appointment, and reviewed indications of each scan in the three following categories. Firstly, for those with alcohol misuse. Secondly, for HCV cases in which Fibroscan® results help decide treatment choice. Thirdly, ‘other’ – for example, consultant discretion due to LFT results.

**Results:**

308 patients were offered Fibroscans® between March 2021 and February 2023.

238 patients attended their appointments, of which 194 were for alcohol misuse, 43 for HCV and 1 ‘other’.

70 patients did not attend their appointments, of which 67 were for alcohol misuse and 3 ‘other’.

Scans for HCV were completed ad hoc (i.e. without an arranged appointment) so are not included in attendance rates. The attendance rate for *scheduled* Fibroscan® appointments (for alcohol misuse and ‘other’) was 74%.

Of the 194 patients scanned for alcohol misuse, 40 were then referred to hepatology with likely cirrhosis.

**Conclusion:**

238 patients underwent a Fibroscan®, leading to 40 hepatology referrals for likely cirrhosis, and 43 patients being offered appropriate HCV treatment.

Crude DNA rates appear greatly improved – 74% attendance at our Fibroscan® appointments vs 32% attendance at hepatology referral appointments.

